# Oxford Nanopore Technologies RNA sequencing data for investigating epigenetic regulation by phase-variable DNA methyltransferase ModD1 in serogroup B *Neisseria meningitidis*

**DOI:** 10.1128/mra.00207-26

**Published:** 2026-05-18

**Authors:** Alice Ascari, Luke V. Blakeway, Calum J. Walsh, Louise M. Judd, Michael P. Jennings, Kate L. Seib

**Affiliations:** 1 Institute for Biomedicine and Glycomics, Griffith University, Gold Coast, Queensland, Australia; 2 Centre for Pathogen Genomics, The Peter Doherty Institute for Infection and Immunity, The University of Melbourne, Parkville, Victoria, Australia; Nanchang University, Nanchang, Jiangxi, China

**Keywords:** *Neisseria meningitidis*, epigenetic regulation, phase variation, DNA methylation, DNA methyltransferase, pathogenic *Neisseria*, gene regulation, Mod

## Abstract

*Neisseria meningitidis* is a primary cause of bacterial meningitis and septicemia, which is a significant global public health concern. Here, we report Oxford Nanopore Technologies RNA sequencing for *N. meningitidis* serogroup B strain M0579. This strain encodes the phase-variable DNA methyltransferase ModD1, an epigenetic regulator associated with hyperinvasive meningococcal B lineages.

## ANNOUNCEMENT

*Neisseria meningitidis* serogroup B is a leading cause of bacterial meningitis and septicemia (1). This pathogen encodes phase-variable N^6^-adenine DNA methyltransferases (Mods), which undergo ON-OFF switching (phase variation) through hypermutable simple sequence repeat (SSR) tracts (2–4). When expressed, Mods establish genome-wide methylation patterns that reshape transcription, generating coordinated changes in gene expression (phasevarions) (3, 5, 6, 7). Distinct Mods carry divergent target-recognition domains that specify their methylation motifs and unique phasevarions (3, 6). Hyperinvasive meningococcal B strain M0579 (clonal complex cc41/44) encodes ModD1 (3), whose phase variation is driven by mutations within its 5′-ACCGA-3′ SSR tract and regulates gene expression through 5′-CCAGC-3′ motif methylation (6). Here, we announce the release of Oxford Nanopore Technologies (ONT) RNA-seq resources for M0579 profiled in a wild-type ModD1 ON state and an isogenic *modD1::kan* knockout (KO) (3), in biological triplicate (Table 1). Full-length ONT long reads enable resolution of transcript boundaries and operon architectures and support operon-level expression comparisons between the ModD1 ON and KO genotypes.

**TABLE 1:** Per-sample ONT sequencing summary table^*a*^^,^^*b*^

Strain	Genotype	Rep	Reads (*n*)	Yield (bp)	*N*_50_ (bp)	SRA accessions	BioSample accessions
M0579	*modD1* ON	1	1,301,624	617,396,901	512	SRR36929598	SAMN54471611
M0579	*modD1* ON	2	580,816	332,431,691	581	SRR36929599	SAMN54471612
M0579	*modD1* ON	3	348,303	192,935,306	581	SRR36929600	SAMN54471613
M0579	*modD1::kan*	1^*c*^	33,691	17,588,279	608	SRR36929601	SAMN54471614
M0579	*modD1::kan*	2^*c*^	5,974,908	3,149,941,066	545	SRR36929602	SAMN54471615
M0579	*modD1::kan*	3^*c*^	16,266	6,010,975	359	SRR36929603	SAMN54471616

^*a*^ Rep, replicate (biological); *n*, number of reads; and SRA, sequence read archive.

^*b*^ The *N. meningitidis* M0579 genome is 2.27 Mb, with 51.53% GC content and 2,266 annotated genes. M0579 is a clinical strain isolated in 1993 from a patient in the USA presenting with invasive meningococcal disease.

^*c*^ The variable read depth across replicates may impact the power of differential expression analysis; however, this ONT long-read resource complements the paired high-depth Illumina RNA-seq data set described separately (8), and both data sets will be integrated in a forthcoming full-length study.

Strains were grown in 20 mL of Gonococcal broth (9) supplemented with 30 µM desferal (Sigma-Aldrich) to reflect host-associated iron-limited conditions, as used previously for *N. meningitidis* transcriptomics (2). Cultures were grown at 37°C with aeration (200 rpm). Total RNA was extracted from 4 mL of mid-logarithmic (OD_600_ = 0.3) phase cells using TRIzol (ThermoFisher) following the manufacturer’s instructions. Concentration and purity were assessed by UV absorbance, with integrity evaluated by agarose gel electrophoresis. The ModD1 ON state of cultured cells was verified (~90% ON) prior to RNA extraction by fluorescent PCR amplification and fragment length analysis of the *modD1* SSR tract, as previously described (3). Following ribosomal RNA depletion (NEBNext rRNA-Depletion Kit, Bacteria; NEB) and polyadenylation [*E. coli* Poly(A)-Polymerase; NEB], full-length double-stranded cDNA was synthesized by PCR (14 cycles) and used for ONT-seq library preparation (cDNA-PCR Barcoding Kit-24-V14; SQK-PCB114.24). Libraries were sequenced on an ONT MinION (R10.4.1 flow cells), and raw signal was basecalled with Dorado v7.6.8 (super-accurate model v4.3.0). Adapter trimming and quality filtering were performed during basecalling (Dorado; --trim all and Q10), generating FASTQ files. *De novo* transcriptome assembly was performed using Flye (v2.9.2; *--nano-hq* preset; genome size-estimate 2.3 Mbp). For reference-based analyses, reads were aligned to the M0579 genome (RefSeq accession: GCF_001029835.1) using minimap2 (v2.26; *-x-map-ont* parameter). Alignment files were processed using SAMtools (v1.21).

Transcriptome reconstruction and quantification were conducted using StringTie2 (v2.2.1). Initial assemblies were generated per genotype using the *-L* long-read flag, guided by reference annotations. Genotype-specific transcriptomes were merged (stringtie; *--merge*), gene- and transcript-level abundance matrices were generated (prepDE.py3; median read length 534 bp determined via seqkit v2.11.0 [10]), and gene counts were independently determined from aligned files (featureCounts; Subread suite v2.0.4) targeting the gene_id attribute. This pipeline was performed on individual and pooled FASTQ files for ON and KO to facilitate sample- and genotype-level comparisons.

Collectively, these ONT long-read RNA-seq data provide an operon-resolved transcriptomics resource that extends beyond differential gene expression analysis, supporting robust genotype comparisons of transcript architecture and mRNA isoforms. This facilitates deeper interrogation of ModD1-driven gene regulation and the molecular mechanism facilitating epigenetic switching with meningococcal transcriptional programs, which will be reported in subsequent manuscripts.

## Data Availability

ONT RNA-seq FASTQ files have been deposited to NCBI under BioProject accession PRJNA1406450 and sequence read archive (SRA) run accessions SRR36929598–SRR36929603 for ModD1 ON and KO replicates, respectively. Associated BioSample accessions for ModD1 ON and KO replicate samples are SAMN54771611–SAMN54771616.
